# GIS Software Package “Epidemiological Atlas of Russia” on Current Infectious Diseases

**DOI:** 10.17691/stm2023.15.6.03

**Published:** 2023-12-27

**Authors:** S.A. Sarskov, M.V. Vyushkov, A.V. Polyanina, S.L. Slavin, N.N. Zaitseva

**Affiliations:** Researcher, Laboratory of GIS Technologies and Bioinformatics; Academician I.N. Blokhina Nizhny Novgorod Scientific Research Institute of Epidemiology and Microbiology of Rospotrebnadzor (Russian Federal Consumer Rights Protection and Human Health Control Service), 71 Malaya Yamskaya St., Nizhny Novgorod, 603950, R; Researcher, Laboratory of GIS Technologies and Bioinformatics; Academician I.N. Blokhina Nizhny Novgorod Scientific Research Institute of Epidemiology and Microbiology of Rospotrebnadzor (Russian Federal Consumer Rights Protection and Human Health Control Service), 71 Malaya Yamskaya St., Nizhny Novgorod, 603950, R; MD, PhD, Senior Researcher, Head of the Laboratory of Viral Hepatitis Epidemiology; Academician I.N. Blokhina Nizhny Novgorod Scientific Research Institute of Epidemiology and Microbiology of Rospotrebnadzor (Russian Federal Consumer Rights Protection and Human Health Control Service), 71 Malaya Yamskaya St., Nizhny Novgorod, 603950, R; Student, Department of Clinical Medicine; Lobachevsky State University of Nizhny Novgorod, 23 Prospekt Gagarina, Nizhny Novgorod, 603022, Russia; MD, DSc, Director; Academician I.N. Blokhina Nizhny Novgorod Scientific Research Institute of Epidemiology and Microbiology of Rospotrebnadzor (Russian Federal Consumer Rights Protection and Human Health Control Service), 71 Malaya Yamskaya St., Nizhny Novgorod, 603950, R

**Keywords:** analysis of infectious/somatic morbidity, GIS technologies, geoinformation systems in epidemiology, database structure, algorithms for calculating epidemiological indicators

## Abstract

**The aim of the study** is to develop a GIS software package “Epidemiological Atlas of Russia” on topical infectious and parasitic diseases in the Russian Federation to create an open and publicly accessible information resource allowing to improve the quality of morbidity epidemiological monitoring and analysis.

**Materials and Methods:**

The GIS software package “Epidemiological Atlas of Russia” was designed for data monitoring, epidemiological analysis, and cartographic visualization and was implemented as a web resource consisting of a web application, a package administration module, and a database management system. The following development tools were used to create the package: JavaScript, PHP, additional mapping libraries (Leaflet, OpenStreetMap), MySQL database management systems, Visual Basic .NET. The primary information for the database was taken from official federal statistical observation forms No.1 and No.2 “Information on infectious and parasitic diseases”.

**Results:**

Analytical methods and GIS technologies used in epidemiological practice were evaluated, optimal technical solutions based on the experience of developing the “Epidemiological Atlas of the Volga Federal District” were selected. A versatile database structure was designed and developed to create an array of input and output statistical values of an epidemiological nature. Original algorithms were created to obtain and evaluate epidemiological indicators. Web application “Epidemiological Atlas of Russia” was developed to present, analyze, and visualize information on infectious and parasitic diseases in the subjects of a district, federal districts, and the Russian Federation as a whole. It allows to work with report forms of the Ministry of Health to organize federal statistical monitoring in the field of health protection and with laboratory studies results to create thematic modules providing detailed information on individual nosologies. Initial data were temporally broken down by months, and spatially, by Russian Federation subjects. All visualization results were dynamically updated and generated based on user’s interactive request.

**Conclusion:**

GIS software package “Epidemiological Atlas of Russia” was developed as an open and publicly accessible information resource and is designed to improve the quality of epidemiological monitoring, operational and retrospective epidemiological analysis of the incidence of current infectious and parasitic diseases in the Russian Federation. The package is intended for use in federal executive authorities, in supervisory authorities and institutions of Rospotrebnadzor, in medical organizations of the Ministry of Health of the Russian Federation and is in line with the state policy aimed to introduce modern technologies into practice.

## Introduction

Epidemiological monitoring of morbidity at the present stage requires not only to collect and analyze data, but also to develop an algorithm capable to use new information and analytical tools. One of the most relevant ways to process epidemiological information, to improve the accuracy and clarity of epidemiological analysis results is the use of geographic information systems (GIS) [1]. GIS is a versatile way of accumulating and storing information allowing to mathematically process the data obtained, displaying them by means of cartographic visualization. The main advantages of geoinformation technologies applied to epidemiological practice are the use of a digital objective data format with clear periodic updating; advanced and convenient epidemiological analysis; scientific justification of proposals to executive authorities allowing to take prompt decisions and to arrange preventive and anti-epidemic measures timely. With wide experience in development and application of GIS “Epidemiological Atlas of the Volga Federal District” at the Academician I.N. Blokhina Nizhny Novgorod Scientific Research Institute of Epidemiology and Microbiology of Rospotrebnadzor (Russia), the creation of a GIS software package on topical infectious and parasitic diseases in the Russian Federation scale and its further improvement (introducing report forms of the Ministry of Health and laboratory research results, improving the quality of monitoring and epidemiological morbidity analysis by expanding analytical functions) is a promising scientific direction able to create a multifunctional informative tool for specialists in the field of monitoring and objective analysis of the epidemiological situation, as well as to take prompt management decisions.

**The aim of the study** is to develop a GIS software package on topical infectious and parasitic diseases in the Russian Federation to create an open and publicly accessible information resource allowing to improve the quality of morbidity epidemiological monitoring and analysis.

## Materials and Methods

To achieve the research aim, the following tasks were solved:

choice of optimal technical solutions based on the experience of developing the “Epidemiological Atlas of the Volga Federal District”;
development of a versatile database structure;
development of algorithms to obtain and evaluate epidemiological indicators;
development of a web application, a package administration module;
testing the package performance.

### Initial data

The data used at GIS “Epidemiological Atlas of Russia” are:

files of federal statistical observation forms No.1 and No.2 “Information on infectious and parasitic diseases” approved by Rosstat Order No.867 dated December 30, 2020 “On approval of federal statistical observation forms with instructions on how to fill them in to organize sanitary condition federal statistical monitoring of the Russian Federation subject by the Federal Service for Supervision of Consumer Rights Protection and Human Welfare” [2];
names and codes of the federal districts and the Russian Federation subjects of each district;
coordinates of the turning points of the borders of federal districts and the Russian Federation subjects corresponding to the administrative division of the Russian Federation, the selected coordinate system and cartographic basis;
names and codes of nosologies corresponding to federal statistical observation forms No.1 and No.2;
data on population number and age distribution in the context of the Russian Federation subjects (data obtained on the basis of reverse recalculation of relative morbidity rates indicated in Rospotrebnadzor analytical tables);
calculations of statistical indicators used in epidemiological analysis;
spatially linked information, including additional characteristics of territories (transport routes, geology, weather data, etc.).

### Database formation

The database is controlled by MySQL free relational database management system. To develop the database structure, the following principles were applied: a database table was created for each federal statistical observation report form No.1 and No.2; all data tables have the same structure to achieve versatility and continuity between the system levels; field names with the same feature (value, content, meaning) are duplicated in all tables, if possible, to write codes and create queries more conveniently.

### Web application and package administration module

A set of software tools for information processing was used to implement the web application of the GIS software package:

HTML (HyperText Markup Language) is a standardized document markup language for viewing web pages in a browser;
CSS is a formal language for describing the appearance of a document;
JavaScript is the programming language most widely used in browsers as a scripting language to make Web pages interactive;
PHP is a general-purpose programming language intensively used to develop web applications and to operate databases and process user requests on web servers;
OpenStreetMap module is a detailed world geographical map distributed on a free license base;
Leaflet Library is an open source mapping library;
DataTables is a component allowing to display data as scrollable and sortable tables distributed on a free license base;
Highcharts is a library to create graphs and diagrams written in JavaScript allowing to easily add interactive, animated graphs to a web application;
jQuery is a JavaScript library designed to simplify bypass and manipulation of HTML DOM tree, to process Ajax events allowing to send and receive data without reloading a web page.

Web development included all work standard stages: graphic design development of the site’s home page and its main pages; creation and testing of the site’s functional part using a program code; the site placement on the Internet and filling with content (epidemiological information). The package administration module was implemented using the object-oriented programming language Visual Basic .NET. To develop the package, standard techniques were used to increase its performance like running most functions in parallel mode, consideration of request processing order.

No information violating anyone’s privacy was used in this work. The study was performed without the participation of humans or animals.

## Results

### Database

The developed database of the “Epidemiological Atlas of Russia” contains spatial and retrospective data on infectious and parasitic morbidity in the country and background information on territories, nosologies, and contingents. The database consists of three table groups.

The first group includes tables generated once and containing reference information on subjects and nosologies. Tables were edited only when report forms, subject composition or structure were changed, or additional statistical indicators were introduced. This table group allowed to: normalize the work of the existing GIS SQL queries; calculate epidemiological indicators; generate tables containing information on nosology incidence and the territory of interest to the user for the selected or entire available year intervals; generate spatial distribution maps of the incidence rate in the studied area or various diagrams for a certain period of time; receive information on nosologies and normative documents regulating their processing through active links to external information resources; work with texts of existing normative documents regulating the rules and norms for each nosology, create samples by disease groups.

The other two groups are tables with accumulated information necessary to create an array of epidemiological output statistical values. The second group consists of tables on the population of municipalities and the Russian Federation subjects supplemented annually with new data, tables of calculated values of long-term average annual morbidity levels, data on infectious and parasitic morbidity as per federal statistical observation form No.2. The third group included tables of infectious and parasitic morbidity, supplemented monthly with new data as per federal statistical observation form No.1.

Advantages of the proposed database version:

the database structure is versatile, not dependent on the district number of subjects, allows to change subjects composition or structure;
the table layout requires no revision when changing the report forms;
a designer of tabular report forms is available allowing to introduce changes based on parameters selected by the user;
practically unlimited number of options to create analytical tables;
expanded set of indicators for in-depth epidemiological analysis;
a new database structure organization was applied; it is versatile and can be used for any project where tabular forms of federal statistical observation are used.

### Package administration module

This module is designed to keep the administrator up-to-date with normative and reference, conditionally permanent, operational, and archived database information, and to differentiate user access to database and software package various components.

The main tasks of the module are:

data reception and control;
database occupancy analysis;
calculation of long term average annual levels;
data export;
maintenance of information certificates;
system check and configuration;
system parameters control;
system parameters update;
MySQL management system maintenance;
database backup;
package reinstall and restore if necessary.

### Data presentation and analysis

**Figure 1.**
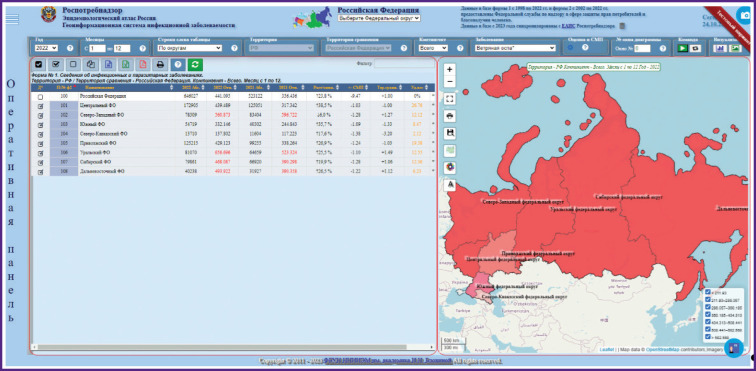
Example of applying the “Epidemiological Atlas of Russia” to the query subject “Incidence of chickenpox in the Russian Federation districts in 2022”

**Figure 2.**
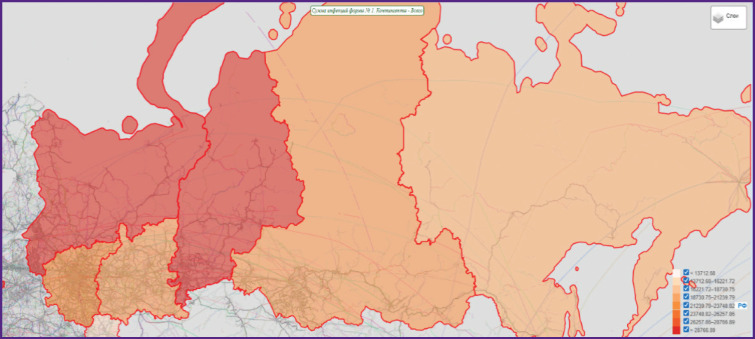
Example of applying the “Epidemiological Atlas of Russia” to the query subject “Incidence of COVID-19 in the Russian Federation districts in 2022”

**Figure 3.**
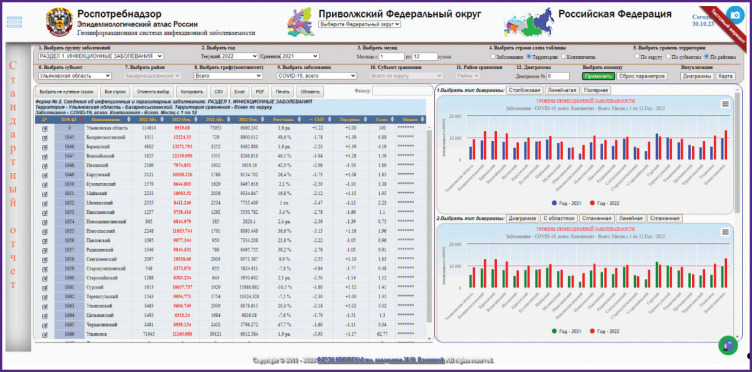
Example of applying the “Epidemiological Atlas of Russia” with the Volga Federal District subsystem to the query subject “Incidence of COVID-19 in Ulyanovsk oblast regions in 2022”

The main way to present data is to visualize epidemiological information as various interactive tables, graphs, and maps, followed by automatic or manual zoning of territories, ranking of infections or case contingents by attribute (incidence rate, long term average annual level, specific gravity, etc.). A web application with advanced functionality and interactive elements assumes close interaction with the user, receiving various parameters from the user for processing and providing results, allows the user not only to view, but also to work with information on the page. Initial data are temporally broken down by months, and spatially, by Russian Federation subjects. The maps show logical sets of epidemiological information with spatial reference, all visualization results are each time dynamically updated and generated based on a query (Figures 1–3).

To date, the following features have been implemented into GIS software package “Epidemiological Atlas of Russia”:

generation of cartographic and tabular materials based on queries to the database, interactive selection of report parameters;
analysis of epidemiological data (classification of territories according to the selected attribute indicator, database sampling for the selected area, assessment of available data completeness, analysis of intra-annual and long term annual dynamics, etc.);
advanced epidemiological analysis, including calculations of seasonal coefficients, determination of actual and threshold morbidity levels, creation of short- and long-term forecasts of morbidity using linear regression, etc.;
cartographic visualization using additional spatially linked information;
export (print) of data graphical representation (schematic maps, graphs, diagrams) and tabular data in XLS, PDF, and CSV formats;
information and reference support on nosologies and normative documents regulating the work through active links to external information resources.

Main GIS module “Epidemiological Atlas of Russia” web application (program) passed the state registration procedure, the registration department of computer programs, databases and topologies of integrated circuits of the Federal Institute of Industrial Property of the Federal Service for Intellectual Property (Moscow, Russia) satisfied the application, and the Certificate of state registration No.2022684310 dated December 13, 2022 was received.

## Discussion

In recent years, regional or local epidemiological atlases became relevant in the practice of sanitary and epidemiological surveillance in the Russian Federation [3–5]. Similar works are far from being rare in the epidemiological surveillance of specific infections. Epidemiological monitoring using GIS technologies exist for vectors of Dengue fever, Chikungunya fever, Zika, for incidence of leptospirosis, plague, brucellosis, etc. All these works combine unavailability of information on morbidity by age groups to search for risk groups, coverage of narrow areas, inability to transfer these works to other infections due to an individual approach and conventional signs fragmentation. As a result, these works cannot be used to build a GIS system that combines the maximum possible list of infections. In some cases, information systems of a larger scale are used, e.g. within the framework of an entire country [6–8]. However, these works are not centralized systems combining the maximum possible list of infections with an expanded analytical and interactive apparatus, and therefore they allow no effective management tasks in the field of public health protection in the Russian Federation in this way. To date, no implemented projects exist for the presentation, analysis, and cartographic visualization of somatic morbidity for the organization of surveillance in the field of public health protection in the Russian Federation.

To develop a geographically distributed GIS software package on a national scale, the tasks were solved of choosing technical solutions for the package being created, determining the structure of input and output data, determining program development phases, stages, and deadlines. Based on a multi-criteria assessment of portal project user interfaces, a system was developed of criteria to evaluate interfaces of problem-oriented web GIS of medical and epidemiological purposes to be applied in web application interface development [9]. Current regulatory documents and literature data are analyzed to identify existing classifications by nosology. The created web application and the package administration module to present and visualize information on infectious and parasitic diseases were tested according to the developed technique [10].

GIS software package “Epidemiological Atlas of Russia” developed on the basis of the Academician I.N. Blokhina Nizhny Novgorod Scientific Research Institute of Epidemiology and Microbiology of Rospotrebnadzor is a unique development accumulating data on all nosological forms of infectious and parasitic morbidity at the Russian Federation subjects. To date, the database contains information on all diseases reflected in the forms of federal statistical observation No.1 and No.2 “Information on Infectious and Parasitic Diseases” (for 1998–2022). This package with advanced functionality and interactive elements involving close interaction with the user, receiving various parameters from the user, processing them and providing results, can become a multifunctional informative tool for specialists in the field of epidemiological situation monitoring and analysis in order to take prompt management decisions during preventive and anti-epidemic measures.

If spatial detailing of epidemiological information to district level is necessary, the software package allows to replicate the subsystem for each district. To date, based on this software package, a new version of the “Epidemiological Atlas of the Volga Federal District” was implemented with accumulated data on infectious and parasitic morbidity in 14 subjects of the Volga Federal District with spatial resolution up to administrative region.

The software package versatility of the database structure has the potential to expand the list of diseases. Introduction into GIS “Epidemiological Atlas of Russia” of the Ministry of Health report forms (No.7 “Information on malignant neoplasms”; No.12 “Information on the number of diseases registered in patients living within a medical organization service area”; No.32 “Information on medical care for pregnant women, women in labor and maternity hospitals”, etc.) will allow:

to improve the quality of epidemiological analysis and monitoring of somatic morbidity in the context of a specific area and the Russian Federation as a whole;
to identify the features and prerequisites for certain nosological forms spread, take prompt management decisions in order to reduce morbidity, improve the population quality and life expectancy, reduce mortality and lethality from somatic diseases;
to calculate short-term and long-term trends in infectious, parasitic, and somatic morbidity;
to optimize interdepartmental interaction between Rospotrebnadzor bodies/organizations and the Ministry of Health, mutually beneficial cooperation in order to monitor and control the population health of different age groups and improve professional competencies of relevant organizations employees.

## Conclusion

GIS software package “Epidemiological Atlas of Russia” developed to create an open and publicly accessible information resource showed its high importance for epidemiological monitoring quality optimization and improvement, operational and retrospective epidemiological analysis of topical infectious and parasitic diseases in the context of both individual subjects and the Russian Federation as a whole. This package meets the state policy on modern technologies introduction into practical activities and is intended for use at the Russian Federation federal executive authorities, bodies and organizations of Rospotrebnadzor, the Ministry of Health. The software package has the potential to expand statistical information (somatic morbidity) and can be demanded by doctors of medical institutions in their practical activities on healthcare organization, epidemiological monitoring of socially significant somatic diseases, as well by employees of medical universities in research and teaching practice to build professional competencies.

This package has a number of advantages:

versatility — the database structure can be used for any project where tabular report forms are used, integration is possible with the current Unified Information Analytical System of Rospotrebnadzor (UIAS of Rospotrebnadzor);
modularity — each program subsystem has four main blocks for operation: operational information, advanced epidemiological analysis, time dependent dynamics, and information and reference support;
usability — a dynamic website is available with advanced interactive elements assuming close interaction with the user.

Due to the program versatility and flexibility, this package’s scope of application can be expanded by introducing laboratory research results as thematic modules providing detailed information on individual nosologies.
